# Cartilage Damage and Bone Marrow Edema in Patellofemoral Versus Tibiofemoral Joints in Knee Osteoarthritis: An MRI-Based Comparative Study

**DOI:** 10.7759/cureus.116584

**Published:** 2026-09-20

**Authors:** Sachin Khanduri, Aniket Chugh, Mohsin Ali Khan, Sana, Aastha Agrawal, Nida Yasrab, Nutan Patel, Punyajeet Sardana

**Affiliations:** 1 Radiology, Era's Lucknow Medical College and Hospital, Lucknow, IND; 2 Radiodiagnosis, Era's Lucknow Medical College and Hospital, Lucknow, IND; 3 Research and Development, Era’s Lucknow Medical College and Hospital, Lucknow, IND

**Keywords:** articular cartilage, bone marrow edema, compartmental analysis, magnetic resonance imaging, patellofemoral joint, tibiofemoral joint

## Abstract

Objectives: This study aimed to evaluate and compare the distribution and severity of articular cartilage damage and bone marrow edema (BME) in the patellofemoral joint (PFJ) and tibiofemoral joint (TFJ) of knees with osteoarthritis (OA) using the Siemens MAGNETOM Vida MRI system (Siemens Healthineers, Erlangen, Germany) and to examine their mutual relationship across compartments.

Methods and materials: This cross-sectional observational study enrolled 60 patients older than 50 years with symptomatic knee OA. All patients underwent a 3.0 Tesla MRI with systematic evaluation of 10 TFJ and five PFJ locations. Articular cartilage integrity was scored on an eight-point ordinal scale (0-6) and subchondral BME on a four-point severity scale (0-3). Two blinded radiologists independently assessed images; Spearman's rank correlation coefficient evaluated associations between the studied parameters.

Results: The study cohort had a mean age of 62.92±4.68 years with female predominance (61.7%; n=37). Inter-observer agreement was substantial (κ=0.84-0.91). Mean cartilage damage scores were comparable between PFJ (3.93±1.79) and TFJ (3.75±3.84; p=0.729). BME scores showed no significant compartmental difference (PFJ: 1.78±0.96; TFJ: 2.02±2.49; p=0.450). Robust positive correlations between cartilage damage and BME were identified at all assessed locations (ρ=0.726-1.000; p<0.001). The overall combined Spearman correlation was 0.924 (p<0.001). Elevated body mass index, higher Kellgren-Lawrence (KL) grade, and increased Western Ontario and McMaster Universities Arthritis Index (WOMAC) pain scores were each significantly associated with higher values for both parameters.

Conclusion: Articular cartilage damage and subchondral BME demonstrate strong interdependence and equivalent compartmental distribution in knee OA, reinforcing the paradigm of OA as a whole-joint disease. MRI-based compartmental assessment provides clinically meaningful structural information beyond conventional radiography.

## Introduction

Osteoarthritis (OA) is a leading cause of chronic disability worldwide, representing the fourth most prevalent disabling condition globally [1,2]. Knee OA accounts for approximately 80% of the total OA burden among all joint disorders in adults [3]. Pathologically, the disease involves progressive deterioration of articular cartilage alongside remodelling of subchondral bone, osteophyte formation, synovial inflammation, periarticular muscle weakness, and ligamentous laxity, collectively resulting in debilitating pain, joint stiffness, and restricted mobility [4].

Contemporary understanding positions knee OA as a disorder of the entire joint, driven by intricate biomechanical and biochemical interactions rather than isolated cartilage wear [5,6]. Mechanical overloading, aging, adiposity, prior joint trauma, and genetic predisposition converge to impair chondrocyte homeostasis, disrupting the equilibrium between extracellular matrix synthesis and enzymatic degradation [7]. In early disease, chondrocytes release pro-inflammatory mediators, including interleukin-1β and tumor necrosis factor-α, that upregulate matrix metalloproteinases, accelerating collagen and proteoglycan depletion [8,9].

Subchondral bone marrow edema (BME), visualized as an ill-defined hyperintense signal on fluid-sensitive MRI sequences, reflects an array of underlying microstructural processes, including trabecular microfractures, marrow fibrosis, focal necrosis, and increased subchondral vascularity [10]. Longitudinal investigations have established BME as a dynamic predictor of structural deterioration in knee OA, with lesion size correlating significantly with cartilage loss over time [11,12].

An important yet incompletely resolved question concerns the compartmental distribution of these pathological changes, specifically whether cartilage damage and BME affect the patellofemoral joint (PFJ) and tibiofemoral joint (TFJ) with comparable frequency and severity. Some population-based MRI studies have reported a predilection for isolated PFJ involvement, particularly in the earlier stages of disease [13,14]. Elucidating compartment-specific patterns of structural pathology may provide a rational basis for targeted therapeutic strategies [13]. The present investigation was therefore designed to evaluate and compare the distribution and severity of articular cartilage damage and BME between the PFJ and TFJ using a 3.0-Tesla Siemens MAGNETOM Vida MRI system (Siemens Healthineers, Erlangen, Germany) in patients with symptomatic knee OA. Given that prior reports of isolated PFJ predominance were largely derived from population-based or early-stage cohorts [13,14], we hypothesized that in a clinic-referred population with more advanced, symptomatic disease, cartilage damage and BME would show comparable involvement across the TFJ and PFJ compartments, consistent with a whole-joint disease process rather than an isolated site of predilection.

## Materials and methods

Study design and setting

This cross-sectional observational study was conducted in the Departments of Radiodiagnosis and Orthopaedics at Era's Lucknow Medical College and Hospital, Lucknow, India, over a 24-month period. The study was reported in accordance with the Strengthening the Reporting of Observational Studies in Epidemiology (STROBE) guidelines for cross-sectional studies.

Ethical approval and consent

Approval from the Institutional Ethics Committee of Era's Lucknow Medical College and Hospital was obtained prior to study commencement (Ref. No. ELMCH & H 2025/503). Written informed consent was obtained from all participants following explanation of the study's nature, purpose, and potential risks. The study was conducted in conformity with the ethical principles of the Declaration of Helsinki.

Participants

Patients referred from the orthopaedics department for knee MRI with a clinical diagnosis of knee OA according to the American College of Rheumatology criteria were screened for enrolment [15]. Eligible participants were aged above 50 years. Exclusion criteria were as follows: concurrent systemic inflammatory joint disease; inability to undergo MRI due to contraindication; or prior history of knee trauma, surgical intervention, or other bone-related disease. Patients were enrolled using a consecutive sampling approach, whereby all eligible individuals referred for knee MRI during the study period were invited to participate until the pre-calculated target sample size was reached.

Sample size

Sample size was computed using the following formula: $\mathrm{n} = \left( \mathrm{Z}_{\alpha/2} + \mathrm{Z}_{\beta} \right)^{2} \times \frac{\mathrm{p}_{1}\left(1 - \mathrm{p}_{1}\right) + \mathrm{p}_{2}\left(1 - \mathrm{p}_{2}\right)}{\mathrm{d}^{2}}$, where p1=0.611 and p2=0.389, with 90% statistical power and a 95% confidence interval, yielding a minimum required sample size of 60 patients (N=60) [16].

Clinical assessment

Demographic data and clinical details were recorded at enrolment. Body mass index (BMI) was calculated from measured height and weight. Radiographic severity was graded using the Kellgren-Lawrence (KL) system (Grades I-IV), and pain severity was quantified by the Western Ontario and McMaster Universities Arthritis Index (WOMAC) pain subscale [17,18].

MRI protocol

All MRI examinations were performed on a 3.0-Tesla Siemens MAGNETOM Vida MRI system using a dedicated phased-array knee coil. The examination protocol comprised oblique sagittal, coronal, and axial proton-density-weighted fat-suppressed fast spin-echo (PD-FS-FSE) sequences and a sagittal T1-weighted fast spin-echo (T1-FSE) sequence. The protocol was based on standard knee MRI acquisition parameters, with a slice thickness of 3 mm. The recommended acquisition parameters for the PD-FS sequences were TR/TE of 3570/39 ms, field of view (FOV) of 160 mm, and matrix of 288 × 384, while the T1-weighted sequence was acquired with a TR/TE of 470/13 ms, FOV of 180 mm, and matrix of 358 × 512. A supplementary oblique sagittal multi-echo gradient-echo sequence (TR 529 ms, TE 16 ms, slice thickness 4 mm, matrix 256 × 256) was included for assessment of cartilage morphology.

MRI scoring

Cartilage damage was graded at 10 TFJ locations (anterior, central, and posterior aspects of medial and lateral femoral condyles; anterior and central aspects of medial and lateral tibial plateau) and five PFJ locations (medial and lateral patellar facets, patellar apex, medial and lateral trochlear surfaces). The scoring scale comprised eight categories (0-6): 0 = normal; 1 = normal thickness with increased signal; 2.0 = partial-thickness focal defect <1 cm; 2.5 = full-thickness focal defect <1 cm; 3 = multiple partial-thickness defects; 4 = diffuse partial-thickness loss; 5 = multiple full-thickness defects; 6 = diffuse full-thickness loss [16].

BME was scored at the same locations: 0 = absent; 1 = mild (<25% subregion area); 2 = moderate (25-50%); 3 = severe (≥50%) [16]. Two radiologists blinded to clinical data independently performed all assessments; inter-observer reliability was quantified by Cohen's kappa. Compartmental 'total' scores represent the sum of the mean subregional scores within each compartment (for example, the total TFJ score is the sum of its 10 constituent subregional mean scores), rather than a normalized or weighted composite; this convention was applied consistently for the TFJ, PFJ, and combined 15-location score. In instances of scoring discrepancy between the two radiologists, consensus was reached through joint re-review of the images; cases that remained unresolved were adjudicated by a third, more senior radiologist not involved in the original scoring.

Statistical analysis

Analyses were performed using IBM SPSS Statistics v21.0 (IBM Corporation, Armonk, NY, USA). Paired t-tests compared compartmental scores. Spearman's rank correlation coefficient (ρ) quantified associations between cartilage damage and BME (thresholds: <0.30 weak; 0.30-0.49 mild; 0.50-0.69 moderate; 0.70-0.89 strong; 0.90-0.99 near-perfect). One-way ANOVA examined score differences across BMI and KL grade categories. Two-tailed p<0.05 was considered statistically significant. There were no missing data for any assessed variable across the 60 enrolled patients. Although cartilage damage and BME scores are ordinal in origin, compartmental and combined scores were treated as approximately continuous for the purposes of paired t-test comparison, consistent with prior MRI-based OA literature employing similar composite scoring systems; this assumption is acknowledged as a limitation given the underlying ordinal scale.

## Results

Baseline characteristics

Sixty patients (age range 52-74 years; mean 62.92±4.68 years) were enrolled. Female sex predominated (61.7%; n=37). Mean BMI was in the overweight range, with 58.3% (n=35) classified as overweight or obese. Radiographic grading revealed KL Grade I in 21.7% (n=13), Grade II in 41.7% (n=25), Grade III in 31.7% (n=19), and Grade IV in 5.0% (n=3). Right knee involvement was present in 56.7% (n=34). Mean disease duration was 2.05±1.22 years and mean WOMAC pain score was 11.92±2.81 (range 6-18).

Cartilage damage and BME at TFJ locations

Table 1 presents scores and correlations at TFJ subregions. Within the medial compartment, the posterior aspect of the medial femoral condyle showed the greatest cartilage damage (0.93±1.21) and BME (0.52±0.72) burden, followed by the central (0.64±0.93 and 0.35±0.58) and anterior (0.43±0.81 and 0.28±0.61) aspects; the medial tibial plateau showed a similar anteroposterior pattern (anterior 0.43±0.89, central 0.20±0.66). Strong positive correlations between cartilage damage and BME were identified across all medial compartment locations (ρ=0.798-0.938; p<0.001). Lateral compartment locations showed consistently lower absolute scores (cartilage damage 0.08-0.20; BME 0.02-0.13) than their medial counterparts, indicating a medial-predominant pattern of structural pathology, while still demonstrating strong-to-near-perfect correlations between the two parameters (ρ=0.513-1.000; p<0.001), including a perfect correlation (ρ=1.000) at the central lateral femoral condyle.

**Table 1:** Correlation of articular cartilage damage and bone marrow edema scores at tibiofemoral joint locations. SD, standard deviation; ρ, Spearman's rank correlation coefficient; p-values were derived from Spearman's rank correlation analysis.

Location	Cartilage Damage Score (Mean±SD)	Bone Marrow Edema Score (Mean±SD)	Spearman's ρ	p-value
Medial Femoral Condyle				
Anterior	0.43±0.81	0.28±0.61	0.934	<0.001
Central	0.64±0.93	0.35±0.58	0.861	<0.001
Posterior	0.93±1.21	0.52±0.72	0.938	<0.001
Medial Tibial Plateau				
Anterior	0.43±0.89	0.20±0.58	0.798	<0.001
Central	0.20±0.66	0.28±0.52	0.883	<0.001
Lateral Femoral Condyle				
Anterior	0.08±0.33	0.02±0.13	0.513	<0.001
Central	0.08±0.46	0.05±0.29	1.000	<0.001
Posterior	0.13±0.70	0.07±0.41	0.923	<0.001
Lateral Tibial Plateau				
Anterior	0.20±0.66	0.12±0.42	0.917	<0.001
Central	0.20±0.66	0.13±0.47	0.917	<0.001

On MRI evaluation, cartilage damage of varying grades was identified at different articular surfaces of the knee, with associated BME of varying severity. Representative findings in the tibiofemoral compartment are shown in Figures 1-3.

**Figure 1:**
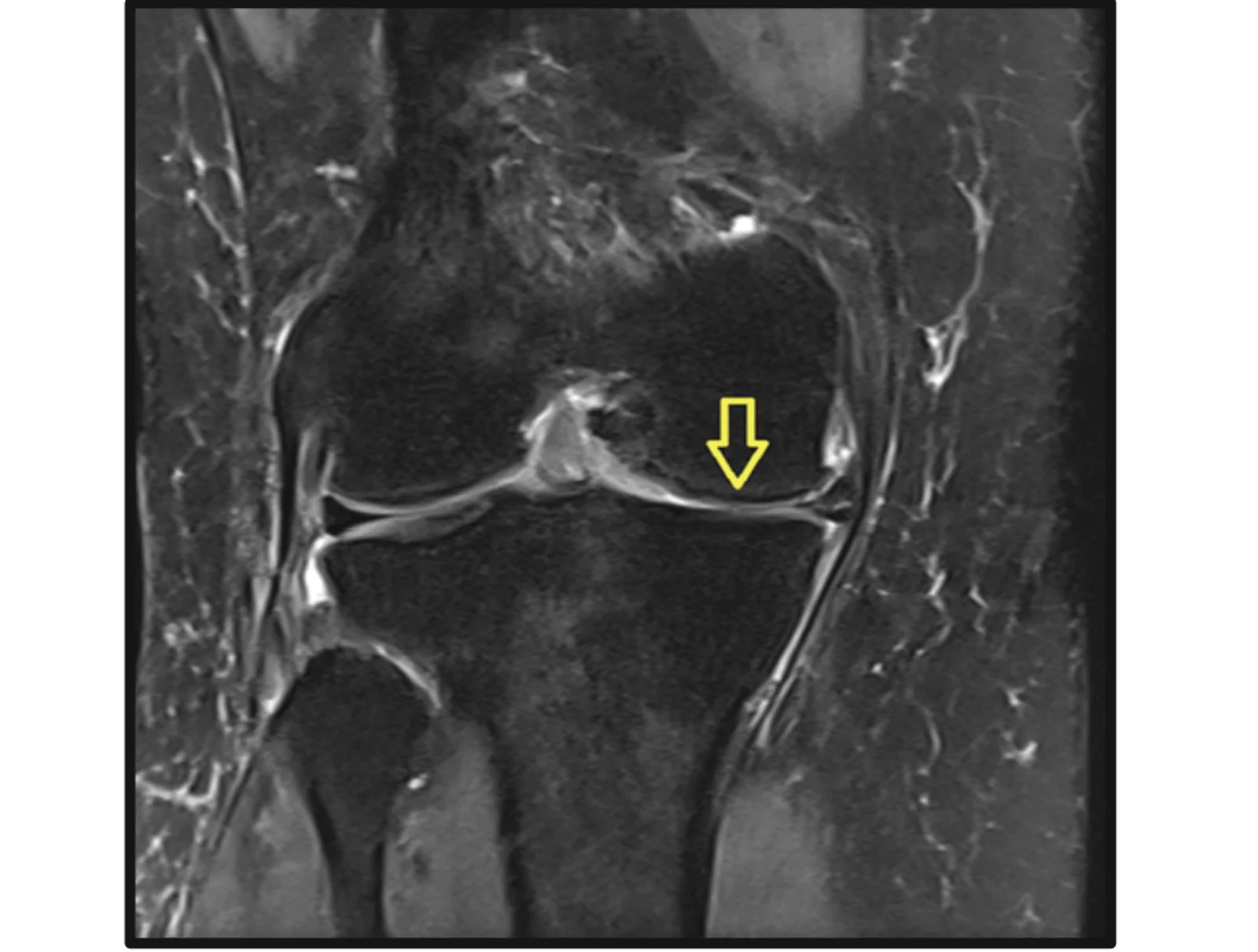
Coronal proton density fat-suppressed MRI image of the knee showing Grade II cartilage damage (yellow arrow) without associated bone marrow edema at the medial femoral condyle.

**Figure 2:**
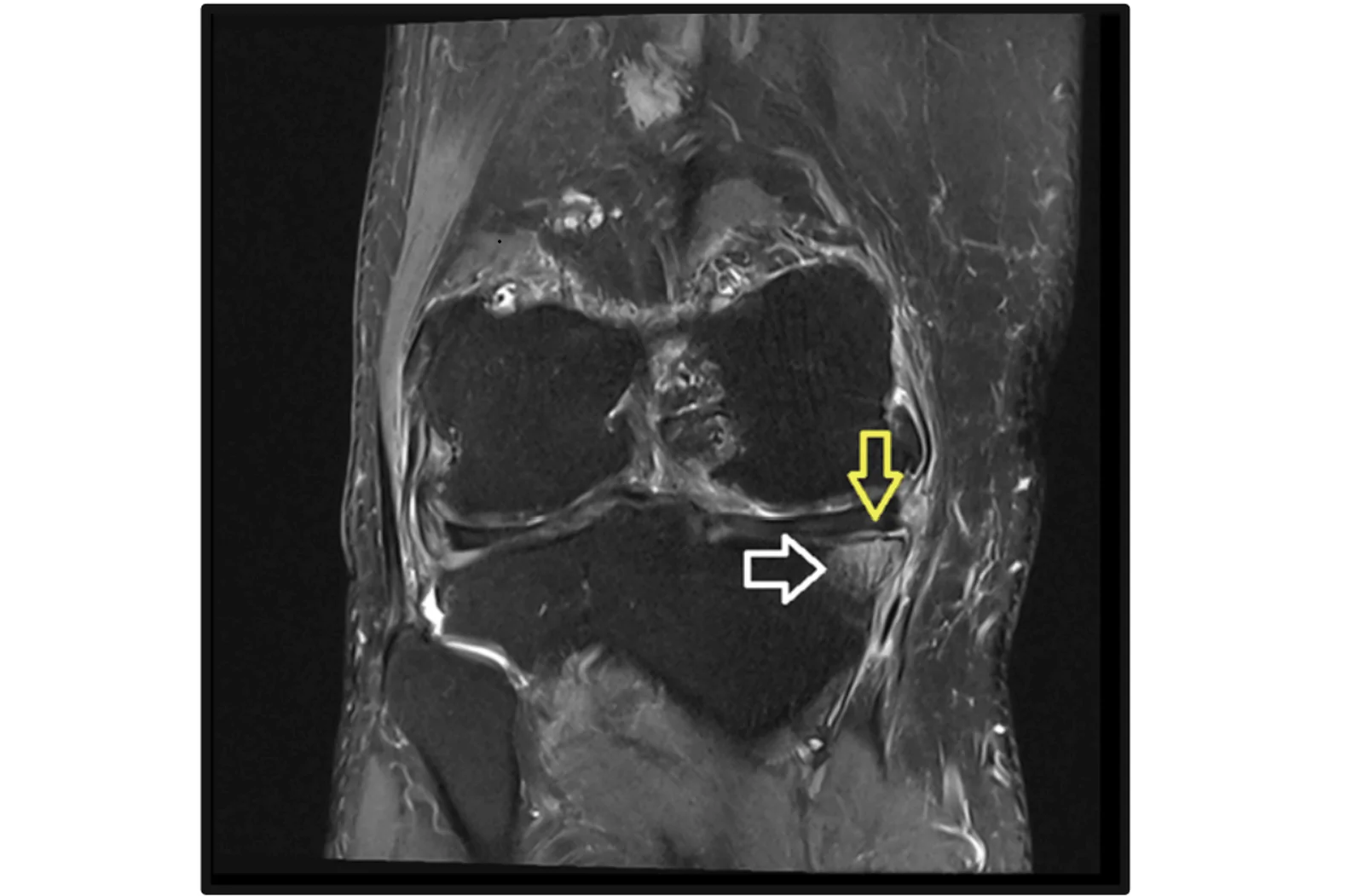
Coronal proton density fat-suppressed MRI image of the knee showing Grade III cartilage damage (yellow arrow) with associated Grade II bone marrow edema (white arrow) at the medial tibial plateau.

**Figure 3:**
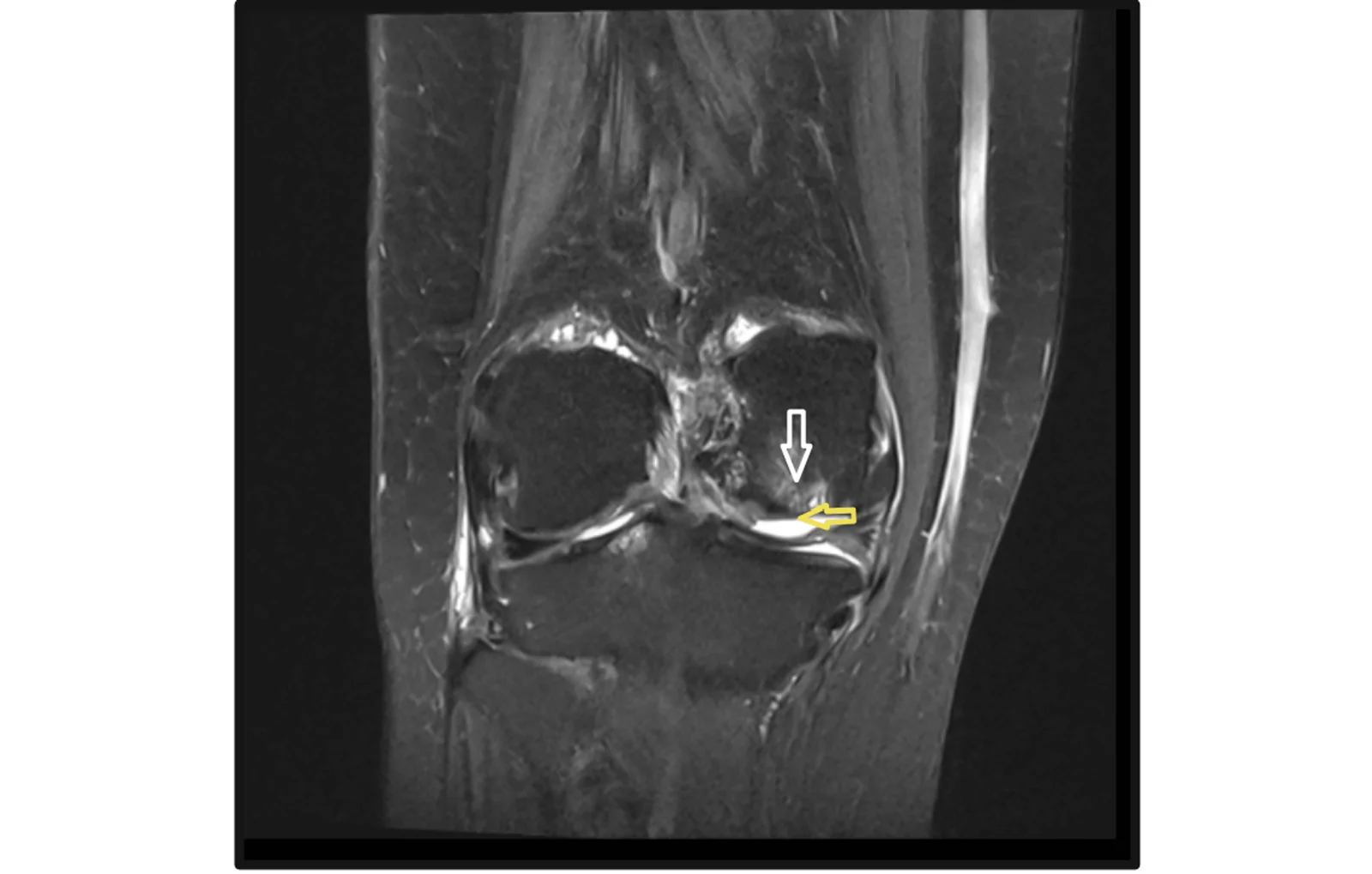
Coronal proton density fat-suppressed MRI image of the knee showing Grade III cartilage damage (yellow arrow) with associated Grade II bone marrow edema (white arrow) at posterior subregion of the medial femoral condyle.

Cartilage damage and BME at PFJ locations

Table 2 presents PFJ subregion values. The medial patellar facet had the highest mean cartilage damage score (1.82±0.97; BME 0.95±0.62) with a strong cartilage-BME correlation (ρ=0.806; p<0.001), followed closely by the lateral patellar facet (cartilage damage 1.56±1.05; BME 0.72±0.67; ρ=0.847; p<0.001). The patellar apex showed a comparatively lower burden (cartilage damage 0.30±0.53; BME 0.12±0.32; ρ=0.635; p<0.001). Trochlear locations recorded the lowest absolute scores within the PFJ (medial trochlea 0.08±0.28 cartilage damage and 0.05±0.22 BME; lateral trochlea 0.17±0.56 and 0.07±0.31, respectively) yet retained strong correlations (medial ρ=0.761; lateral ρ=0.726; both p<0.001), indicating that the patellar facets, rather than the trochlear surfaces, bear the predominant structural burden within the PFJ.

**Table 2:** Correlation of articular cartilage damage and bone marrow edema scores at patellofemoral joint locations. SD, standard deviation; ρ, Spearman's rank correlation coefficient; p-values were derived from Spearman's rank correlation analysis.

Location	Cartilage Damage Score (Mean±SD)	Bone Marrow Edema Score (Mean±SD)	Spearman's ρ	p-value
Patella				
Medial Facet	1.82±0.97	0.95±0.62	0.806	<0.001
Lateral Facet	1.56±1.05	0.72±0.67	0.847	<0.001
Apex	0.30±0.53	0.12±0.32	0.635	<0.001
Trochlea				
Medial	0.08±0.28	0.05±0.22	0.761	<0.001
Lateral	0.17±0.56	0.07±0.31	0.726	<0.001

Representative findings in the patellofemoral compartment are shown in Figures 4-6, demonstrating different grades of cartilage damage with associated BME of varying severity.

**Figure 4:**
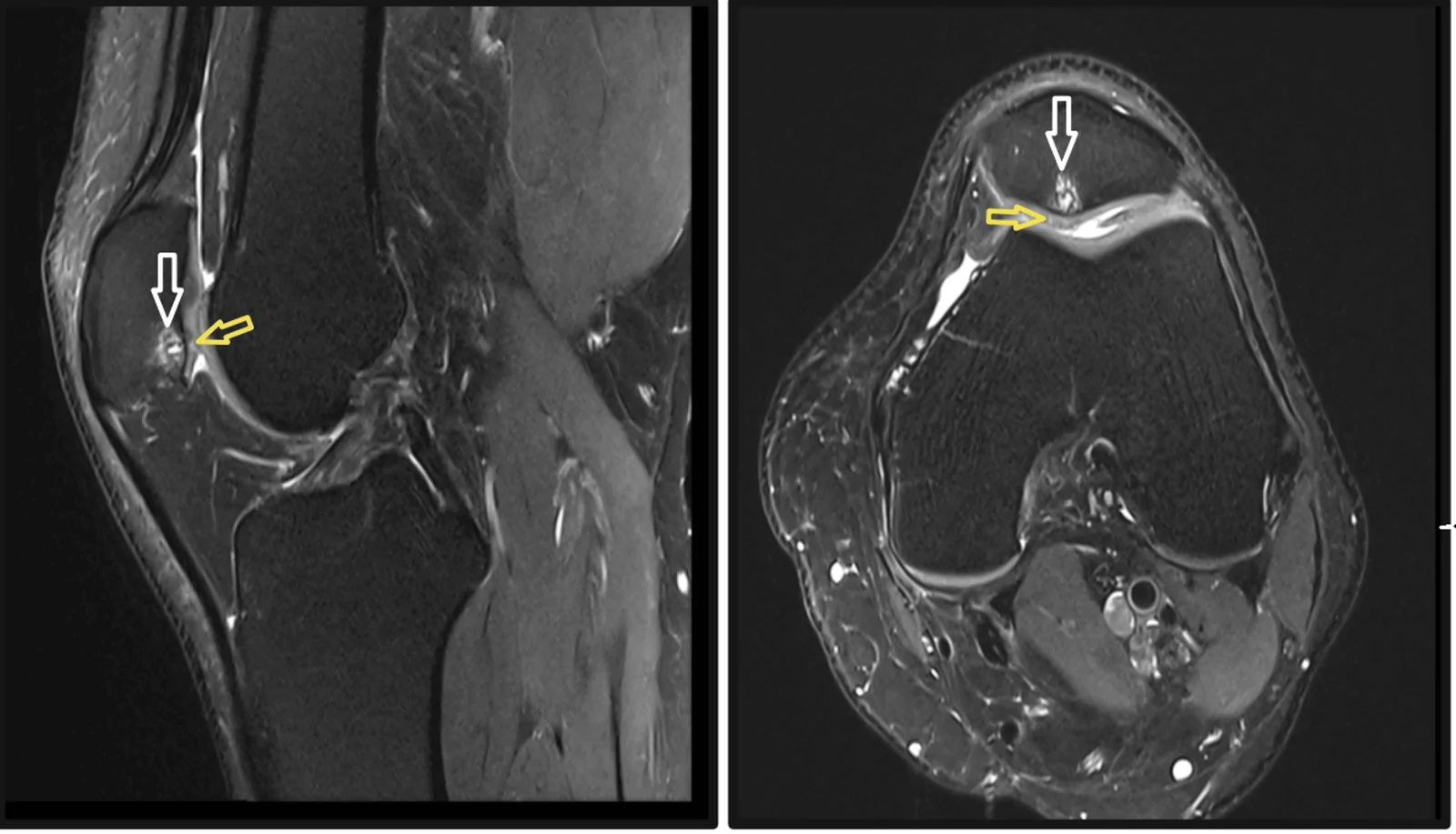
Proton density fat-suppressed sagittal and transverse MRI image of the knee showing Grade I cartilage damage (yellow arrow) with associated Grade I bone marrow edema (white arrow) at the medial facet of the patella.

**Figure 5:**
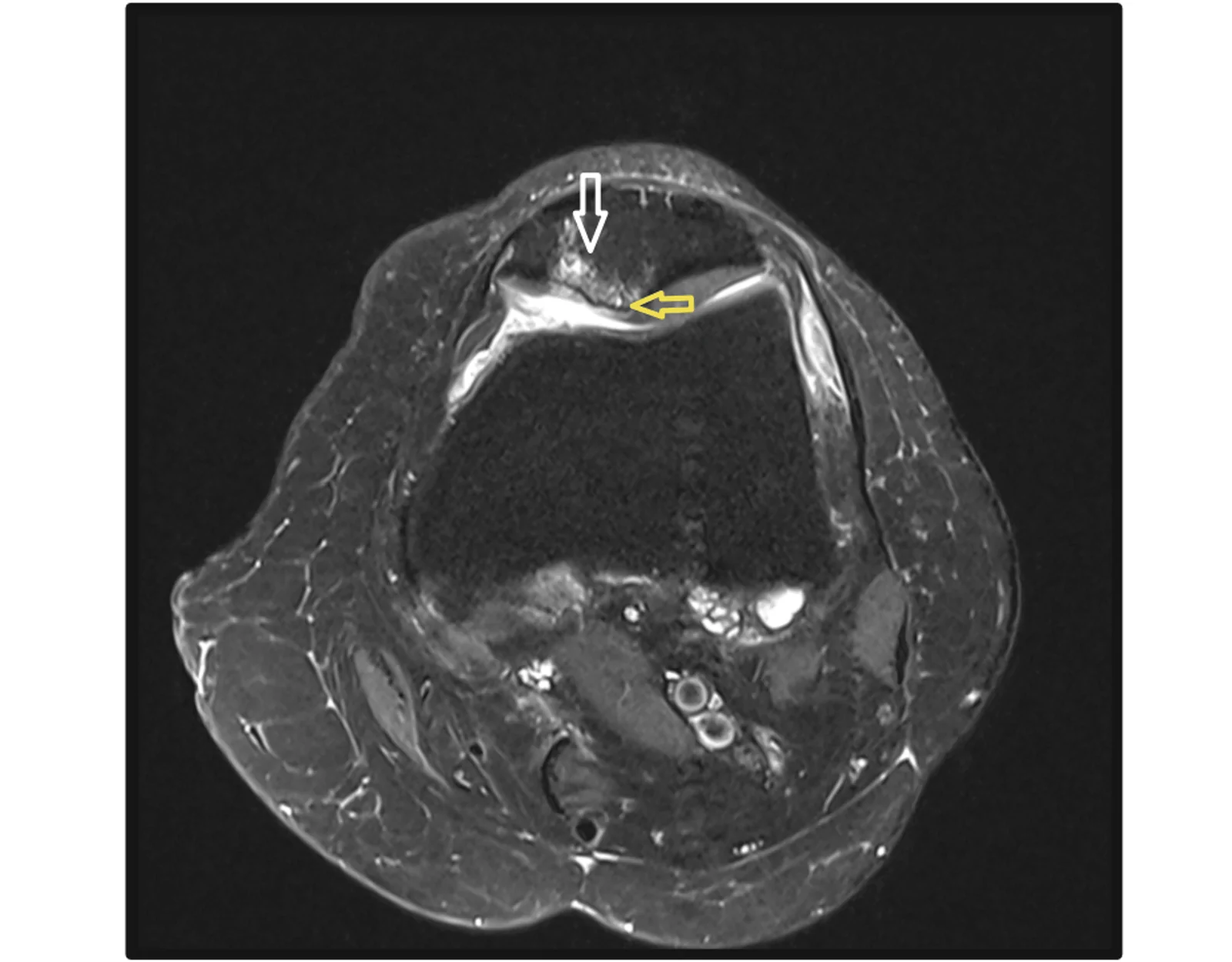
Proton density fat-suppressed transverse MRI image of the knee showing Grade IV cartilage damage (yellow arrow) with associated Grade II bone marrow edema (white arrow) at the medial facet of the patella.

**Figure 6:**
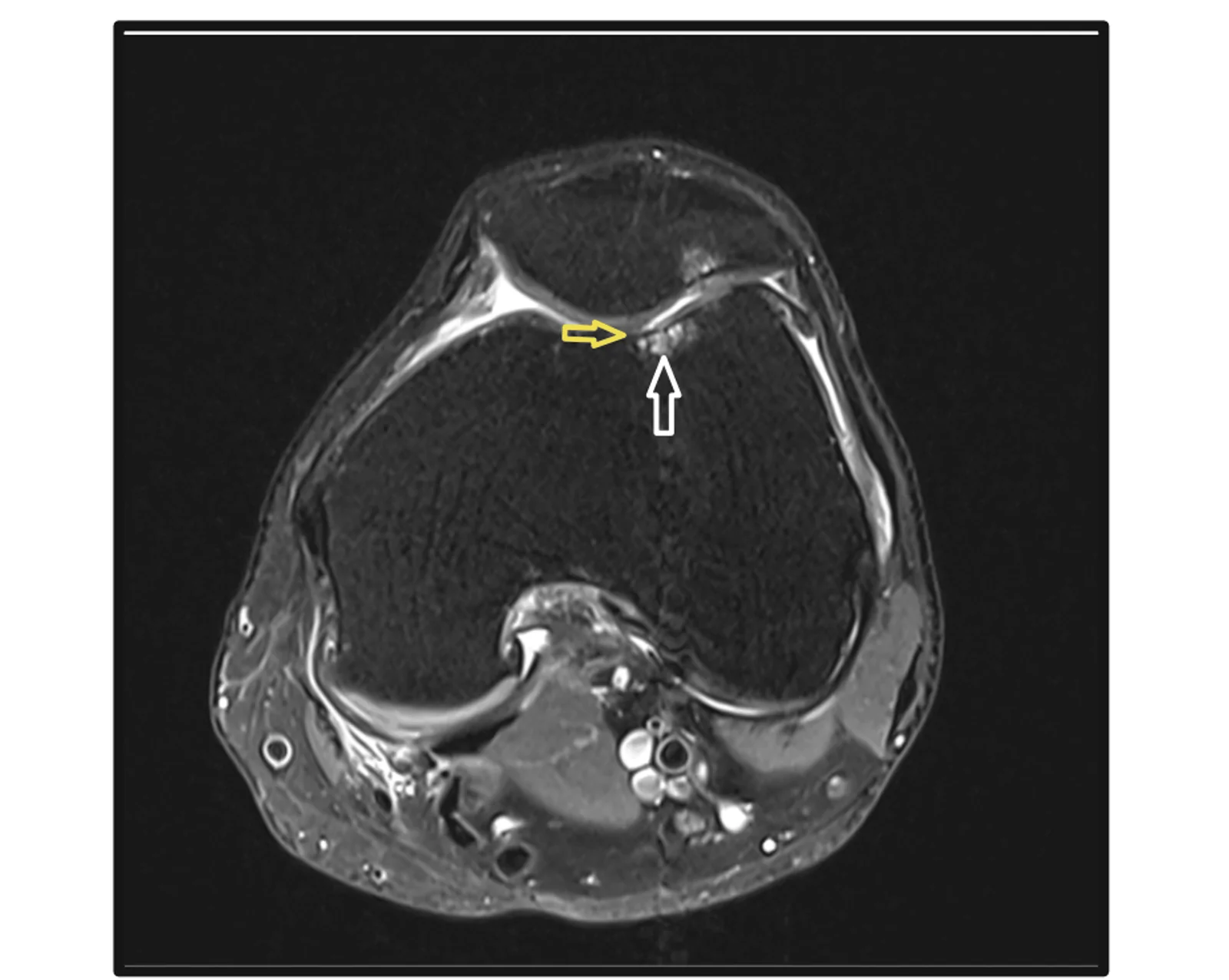
Proton density fat-suppressed transverse MRI image of the knee-patellofemoral joint compartment showing Grade II cartilage damage (yellow arrow) with associated Grade I bone marrow edema (white arrow) at the lateral trochlea.

Total compartmental scores and correlations

Table 3 summarises total locational scores. Within the TFJ, the medial femoral condyle contributed the largest share of the compartmental total (cartilage damage 2.01±2.08; BME 1.15±1.40; ρ=0.913; p<0.001), followed by the medial tibial plateau (1.04±1.41 and 0.48±0.85; ρ=0.851), the lateral tibial plateau (0.40±1.22 and 0.25±0.84; ρ=0.888), and the lateral femoral condyle, which contributed the least (0.30±1.12 and 0.13±0.68; ρ=0.732), consistent with the medial-predominant pattern seen at the subregional level. Overall, the TFJ cartilage damage score was 3.75±3.84 with BME score 2.02±2.49 (ρ=0.956; p<0.001). Within the PFJ, the patella accounted for nearly the entire compartmental burden (cartilage damage 3.68±1.58; BME 1.78±0.96; ρ=0.802; p<0.001), while the trochlea contributed only a small fraction (0.25±0.63 and 0.12±0.37; ρ=0.783). The PFJ cartilage damage score was 3.93±1.79 with BME score 1.78±0.96 (ρ=0.800; p<0.001). The overall combined score across all 15 locations yielded the strongest correlation (ρ=0.924; p<0.001), reinforcing that cartilage damage and BME track closely together regardless of anatomical subregion.

**Table 3:** Total compartmental cartilage damage and bone marrow edema scores and their correlations. SD, standard deviation; n, number of locations assessed per compartment; ρ, Spearman's rank correlation coefficient; p-values were derived from Spearman's rank correlation analysis.

Compartment	Locations (n)	Cartilage Damage Score (Mean±SD)	Bone Marrow Edema Score (Mean±SD)	ρ	p-value
Total Tibiofemoral Joint	10	3.75±3.84	2.02±2.49	0.956	<0.001
Medial Femoral Condyle	3	2.01±2.08	1.15±1.40	0.913	<0.001
Medial Tibial Plateau	2	1.04±1.41	0.48±0.85	0.851	<0.001
Lateral Femoral Condyle	3	0.30±1.12	0.13±0.68	0.732	<0.001
Lateral Tibial Plateau	2	0.40±1.22	0.25±0.84	0.888	<0.001
Total Patellofemoral Joint	5	3.93±1.79	1.78±0.96	0.800	<0.001
Patella	3	3.68±1.58	1.78±0.96	0.802	<0.001
Trochlea	2	0.25±0.63	0.12±0.37	0.783	<0.001
Overall Combined	15	7.68±4.56	3.80±2.94	0.924	<0.001

Compartmental comparison and clinical associations

Table 4 presents the comparison of MRI-derived cartilage damage and BME scores between the TFJ and PFJ. Mean cartilage damage scores were comparable between the TFJ (3.75 ± 3.84) and PFJ (3.93 ± 1.79), with no statistically significant difference (t = 0.349, p = 0.729). Similarly, mean BME scores were comparable between the TFJ (2.02 ± 2.49) and PFJ (1.78 ± 0.96), with no statistically significant difference (t = 0.761, p = 0.450). These findings indicate no evidence of a significant difference in MRI-derived cartilage damage or BME burden between the two compartments. However, the non-significant p-values should not be interpreted as definitive evidence of equivalence. Both cartilage damage and BME scores increased significantly with higher BMI (p < 0.05) and advancing KL grade (p < 0.001), supporting the clinical validity of the MRI scoring system against established measures of disease severity. WOMAC pain scores demonstrated moderate positive correlations with overall cartilage damage (ρ = 0.631) and BME (ρ = 0.590; both p < 0.001), indicating that greater structural abnormalities on MRI were associated with greater patient-reported pain.

**Table 4:** Comparison of cartilage damage and bone marrow edema scores between tibiofemoral and patellofemoral joint compartments. SD, standard deviation; p-values were derived from paired t-tests comparing tibiofemoral and patellofemoral joint scores.

Parameter	Tibiofemoral Joint (Mean±SD)	Patellofemoral Joint (Mean±SD)	t-statistic	p-value
Cartilage Damage Score	3.75±3.84	3.93±1.79	0.349	0.729
Bone Marrow Edema Score	2.02±2.49	1.78±0.96	0.761	0.450

## Discussion

This investigation examined the compartmental distribution of articular cartilage damage and subchondral BME in symptomatic knee OA using high-field 3.0 Tesla MRI and evaluated the quantitative relationship between these two structural parameters. The principal findings were (1) cartilage damage and BME scores did not differ significantly between the PFJ and TFJ; (2) strong-to-near-perfect positive correlations between cartilage damage and BME were present at all assessed locations; and (3) both parameters were significantly associated with increasing BMI, radiographic severity, and pain scores.

The demographic profile of our cohort, mean age 62.92 years and female predominance of 61.7% (n=37), closely mirrors that reported in large multicenter studies. Roemer et al. described a comparable mean age of 62.9 years with 68.1% female participants in the Multicenter Osteoarthritis Study (MOST) cohort [19], while Stefanik et al. documented analogous demographics in their compartmental prevalence analysis [20]. The higher prevalence of knee OA in women after the fifth decade likely reflects hormonal changes after menopause that influence articular cartilage metabolism and subchondral bone remodeling [5].

The strong positive correlations between cartilage damage and BME observed across all subregions (ρ=0.726-1.000) constitute a central finding. This intimate coupling reflects shared pathomechanical pathways: subchondral insufficiency fractures alter the mechanical environment of the overlying cartilage, while cartilage thinning transfers greater loads to the subchondral bone, perpetuating marrow edema in a bidirectional relationship [10, 11]. These observations are consistent with those of Dong et al. [16], Driban et al. [21], and Crema et al. [22]. The composite correlation coefficient of 0.924 obtained in the present study underscores the tight structural interdependence of these pathologies, as visually demonstrated in Figures 1-6.

The absence of a statistically significant difference between TFJ and PFJ (cartilage damage: 3.93 vs 3.75, p=0.729; BME: 1.78 vs 2.02, p=0.450) contrasts with earlier reports identifying preferential PFJ involvement in earlier or asymptomatic cohorts. Stefanik et al. found isolated PFJ damage more prevalent than isolated TFJ damage in a population-based sample [20], and Dong et al. reported a higher cartilage damage burden in the PFJ (relative risk 1.57) [16]. Our clinic-referred cohort predominantly had moderate-to-severe OA (KL grade II-III in 73.4%; n=44), at which point pathological involvement has propagated across both compartments, consistent with the disease propagation model proposed by Stefanik et al. [13].

The significant association between higher BMI and greater severity of both parameters corroborates the established role of mechanical loading in OA pathogenesis. Keng et al. reported that BMI exceeding 25 kg/m² approximately tripled the risk of moderate cartilage damage [23]. Beyond mechanical overloading, adipose tissue-derived adipokines exert direct pro-catabolic effects on chondrocytes and subchondral osteoblasts [5,6]. The significant positive association with advancing KL grade validates MRI-detected structural measures against the radiographic standard while confirming that radiography may underestimate regional pathology compared with MRI [24].

The moderate correlations between WOMAC pain scores and cartilage damage (ρ=0.631) and BME (ρ=0.590) support the clinical significance of these imaging parameters. BME is thought to be a particularly important pain generator in OA, reflecting active subchondral bone stress and nociceptive pathway stimulation [10]. Xiao and Sang similarly confirmed a significant association between BME severity and pain scores in patients with knee OA [25].

Limitations

The cross-sectional design precludes determination of the temporal sequence of cartilage and BME changes. The sample size, though statistically determined, is relatively modest. Single-center recruitment may limit generalizability. The absence of longitudinal follow-up prevents characterization of compartment-specific progression trajectories. Future multicenter prospective studies with serial MRI are warranted.

## Conclusions

This MRI-based comparative study demonstrates that articular cartilage damage and subchondral BME exhibit strong, consistent positive correlations across all 15 assessed tibiofemoral and patellofemoral locations, with the medial tibiofemoral compartment and the patella emerging as the principal sites of structural burden within their respective joints. Neither cartilage damage nor BME scores differed significantly between the tibiofemoral and patellofemoral compartments, suggesting a broadly comparable, whole-joint distribution of disease in this moderate-to-severe, clinic-referred cohort rather than the isolated patellofemoral predominance reported in some earlier, population-based studies of milder disease; as these are non-significant findings, they indicate an absence of evidence for a compartmental difference rather than confirmed statistical equivalence, and should be interpreted accordingly. Both parameters escalated significantly with increasing BMI, radiographic (KL) severity, and WOMAC pain burden, reinforcing the clinical relevance of MRI-detected structural change as a correlate of established measures of OA severity.

Taken together, these findings support the contemporary paradigm of knee OA as a whole-joint disease in which cartilage degeneration and subchondral bone remodelling progress in tandem rather than in isolation, and they suggest that compartment-specific MRI assessment provides clinically meaningful structural information beyond that available from conventional radiography alone. Recognition of this coupled deterioration across both the tibiofemoral and patellofemoral compartments has direct implications for treatment targeting, patient counselling, and disease monitoring, since interventions aimed at only one compartment or one tissue type may fail to address the full structural burden of disease. Given the cross-sectional design of this study, future multicentre, longitudinal investigations with serial MRI are warranted to clarify the temporal sequence of cartilage and bone marrow change, to define compartment-specific progression rates, and to evaluate the prognostic utility of quantitative MRI scoring systems in guiding individualised therapeutic decision-making.
